# Trunk Motion System (TMS) Using Printed Body Worn Sensor (BWS) via Data Fusion Approach

**DOI:** 10.3390/s17010112

**Published:** 2017-01-08

**Authors:** Mohammad Iman Mokhlespour Esfahani, Omid Zobeiri, Behzad Moshiri, Roya Narimani, Mohammad Mehravar, Ehsan Rashedi, Mohamad Parnianpour

**Affiliations:** 1 Department of Industrial and Systems Engineering, Virginia Polytechnic Institute and State University, Blacksburg, VA 24061, USA; 2 Laboratory of Wearable Technologies and Neuromusculoskeletal Research, School of Mechanical Engineering, Sharif University of Technology, Tehran 11155-9567, Iran; narimani@sharif.edu (R.N.); parnianpour@sharif.edu (M.P.); 3 Department of Biomedical Engineering, McGill University, Montréal, QC H3A 2B4, Canada; omid.zobeiri@mail.McGill.ca; 4 Control and Intelligent Processing, Center of Excellence, School of Electrical and Computer Engineering, University of Tehran, Tehran 14395-515, Iran; moshiri@ut.ac.ir; 5 Musculoskeletal Rehabilitation Research Center, Ahvaz Jundishapur University of Medical Sciences, Ahvaz 6135733133, Iran; mohammad.mehravar@gmail.com; 6 Department of Industrial and Systems Engineering, Rochester Institute of Technology, Rochester, NY 14623-5603, USA; exreie@rit.edu

**Keywords:** wearable system, body worn sensor, trunk movement, sensor fusion

## Abstract

Human movement analysis is an important part of biomechanics and rehabilitation, for which many measurement systems are introduced. Among these, wearable devices have substantial biomedical applications, primarily since they can be implemented both in indoor and outdoor applications. In this study, a Trunk Motion System (TMS) using printed Body-Worn Sensors (BWS) is designed and developed. TMS can measure three-dimensional (3D) trunk motions, is lightweight, and is a portable and non-invasive system. After the recognition of sensor locations, twelve BWSs were printed on stretchable clothing with the purpose of measuring the 3D trunk movements. To integrate BWSs data, a neural network data fusion algorithm was used. The outcome of this algorithm along with the actual 3D anatomical movements (obtained by Qualisys system) were used to calibrate the TMS. Three healthy participants with different physical characteristics participated in the calibration tests. Seven different tasks (each repeated three times) were performed, involving five planar, and two multiplanar movements. Results showed that the accuracy of TMS system was less than 1.0°, 0.8°, 0.6°, 0.8°, 0.9°, and 1.3° for flexion/extension, left/right lateral bending, left/right axial rotation, and multi-planar motions, respectively. In addition, the accuracy of TMS for the identified movement was less than 2.7°. TMS, developed to monitor and measure the trunk orientations, can have diverse applications in clinical, biomechanical, and ergonomic studies to prevent musculoskeletal injuries, and to determine the impact of interventions.

## 1. Introduction

Spinal injuries and back pain are common health problems among adults. Davatchi et al. have reported this as a growing challenge in both developing and developed countries [1,2], probably due to the lack of proper prevention methods. To facilitate the prevention of spinal injuries, biomechanical analysis and simulation of human movement (kinematics and kinetics) have been developed, particularly since it is almost impossible to directly measure the spinal loads.

There are several methods and commercial devices used to measure kinematic parameters of the human body. These measurement systems are broadly categorized into two groups: unwearable and wearable systems. Video analysis, optoelectronic analysis, photogrammetry, ultrasound, and electromagnetic tracking systems are among the unwearable systems, while flexible sensors, accelerometers, gyroscopes, magnetometers, textile sensors, and inertial measurement units (IMU) are prominent kinds of wearable devices [3]. Unwearable systems are mainly used in laboratories as they typically require external emitters or cameras. However, wearable systems are portable and can be used in a variety of indoor and outdoor biomedical applications such as fall detection, rehabilitation, ergonomics, and sports biomechanics [3,4,5,6,7,8,9,10].

It has been reported that sensor wearers (e.g., patients and workers) prefer a small, lightweight device that is easily operated and maintained, while also being compatible with daily activities [11,12]. Meanwhile, the preferred position of wearable sensors was the upper limbs; where, for example, users consented to wear them every day for more than 20 h [12]. Therefore, researchers have been recently inclined to develop and utilize portable, non-invasive, low-cost, and lightweight devices in their studies [11,12]. For this purpose, they have primarily selected two wearable systems in the past decade: Inertial Measurement Units (IMUs) and textile sensors.

IMU, an electronic device based on microelectromechanical systems (MEMS), consists of an accelerometer, a gyroscope, and an optional magnetometer [9,13,14,15,16]. It can be placed on body segments of interest, and capture segment kinematics (e.g., 3D acceleration, angular velocity, and angular orientations) in a body-fixed reference frame. For accurate and drift-free measurement, several fusion algorithms have been reported, such as the Kalman filter, combining the different underlying sensors [17,18]. However, IMUs are limited by some environmental effects and data collection issues: (1) environmental magnetic fields and ferromagnetic objects cause disturbances in the output of the magnetometer; (2) the offset error of the gyroscope cannot be completely removed; and (3) temperature can affect its performance [19].

Textile sensors are a well-known technology with many recent advances in the fabric industry. They have been used in diverse areas such as electrocardiography, muscle activity measurements, electroencephalograms, and plethysmography [10,20,21,22,23,24,25,26]. Wearable devices in clothing have also been used for posture and movement analysis [25], and in sports research [26]. For example, Harms et al. [27] used textile sensors to classify upper body postures. Mattmann et al. [28] utilized 21 textile sensors to determine 27 distinct trunk and shoulder movements. Although textile sensors are limited by some environmental effects, using this technology has significant potential advantages. For example, such sensors can be tailored for individuals, they are relatively inexpensive (potentially even disposable), and can be implemented in close proximity to the body [28]. In the present study, a new device called Trunk Motion system (TMS) is presented to record trunk motion using printed textile sensors that are called body worn sensors (BWSs) [29]. Some advantages of the TMS include the use of printed BWSs, non-invasiveness, lightweight, and capability of quantifying 3D trunk movements.

## 2. Materials and Methods

### 2.1. Materials

#### 2.1.1. Body Worn Sensor (BWS)

**Figure 1:**
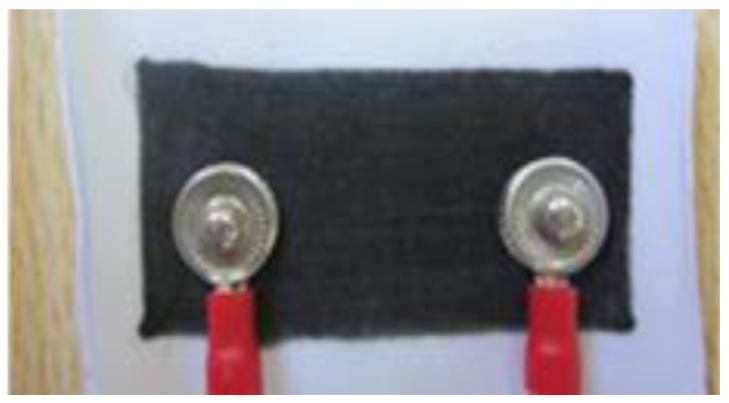
Body Worn Sensor (BWS) used in development of Trunk Motion System (TMS). Metal snap buttons used to create the needed electrical connections between the BWS and lead wires.

BWSs are used to quantify the strain in textile by measuring the resistance changes in nano electroactive conducting polymer between the two ends of BWS [29]. Working as a potentiometer, the resistance range was between 2 kΩ and 70 kΩ, linearly corresponded to no tension and 50% maximum elongation, respectively. The size of BWS was 20 × 40 mm, and its performance was not affected by humidity [29]. Details about manufacturing the BWS can be obtained from our recent study [29]. BWSs are designed to measure strain in textiles, thus, have to be connected to the electronic board through wires and connections. Metal snap buttons were attached to both sides of the BWS to connect the sensor to wires. These connectors are small and easily installable. A prototype of this sensor and its electrical connections between the BWS and lead wires are shown in Figure 1.

#### 2.1.2. Stretchable Shirt

**Figure 2:**
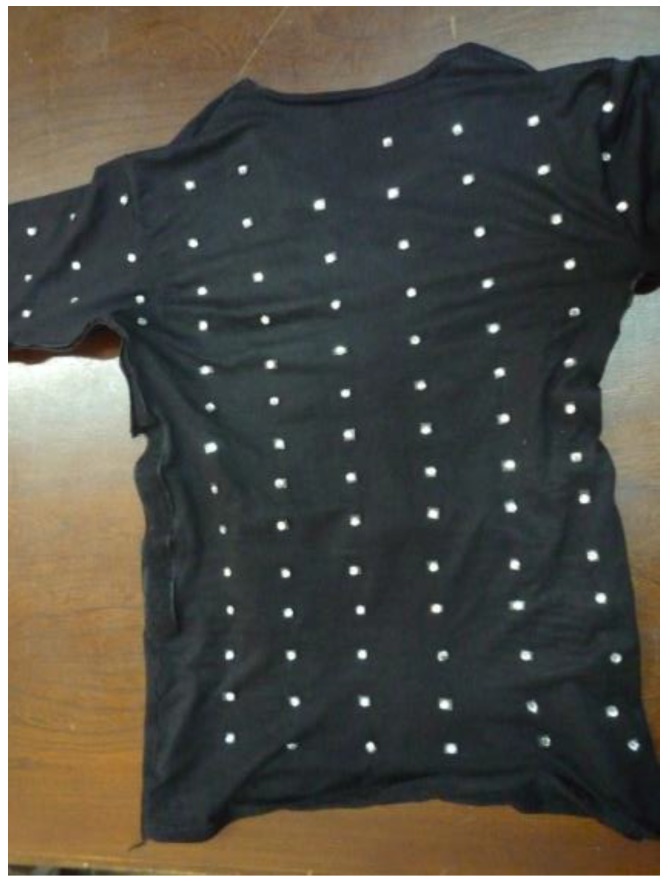
The arrays of spherical markers on the stretchable shirt used to determine sensor locations.

A stretchable shirt was used to determine the possible positions of BWS on clothing. In fact, this shirt could show the direction of largest stretches in different movements, from which the most proper BWS locations could be determined. To achieve this goal, ninety spherical markers were attached to different places of this stretchable shirt (Figure 2). Qualisys system (Qualisys Inc., Gothenburg, Sweden) was used to track the 3D location of these markers.

#### 2.1.3. Measurement Setup

**Figure 3:**
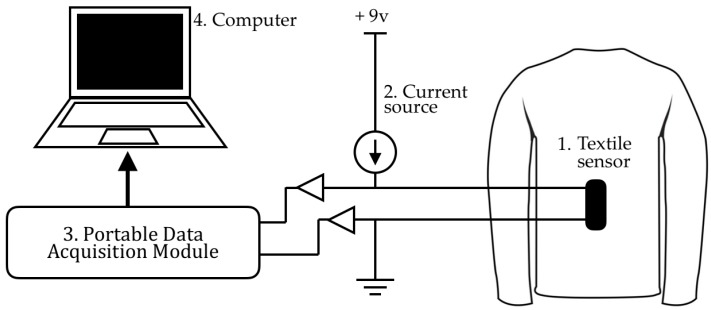
The block diagram of the electronic board and the system configuration as developed for use within TMS. The BWS, electronic board (includes: battery, current source, and potentiometer elements), portable data acquisition module, and computer are numbered 1 to 4, respectively. Due to the difference among sample sensors in their initial resistances, the potentiometer was regulated for each sample such that the BWSs’ signals start from around 1 V.

As mentioned above, BWSs’ functionality is similar to potentiometers. Therefore, an electrical board is needed to convert the resistance changes to corresponding voltage values. This electrical board was designed and developed using current sources, and low pass filter circuits. The current amplitudes were utilized to provide the input voltage range of the analog to digital converter, and a low-pass filter circuit was used to remove the high-frequency noises of the signal. Filtered signals were digitized by a portable data acquisition unit that had an 8-bit analog to digital converter. A microcontroller unit controlled the conversion operation as well as a data transmission to a computer database via a USB port. The block diagram of the electronic board and the system configuration are illustrated in Figure 3.

### 2.2. Methods

#### 2.2.1. Determination of the Sensors’ Position

The placement of wearable device was always challenging for researchers and practitioners. For example, many scientists have used accelerometers for behavior monitoring in diverse segments of the body including the hip, wrist, chest, ankle [30,31,32]. However, they could not find the best placement for the installation of wearable devices to accurately monitor all activities of daily life [33,34]. 

Similarly, the position of textile sensors is an important subject. Some researchers empirically determined the placement of the textile sensors on the garment [35,36,37,38,39]. Several researchers have placed the textile sensors based on the natural anatomical movements of body joints [40,41,42,43,44]. The empirical and anatomical selections might be proper solutions for the past studies because they usually measured or classified the simple body movements. However, complex joints and segments such as trunk and shoulder as well as human activity recognition need a more accurate method to determine the number and placement of textile sensors [45]. Furthermore, Mattmann et al. [28] developed a shirt and arranged the markers on it, then used a motion tracking system to investigate and determine the locations of sensors by measuring the respective strain patterns for 27 distinct trunk and shoulder movements. After that, they utilized 21 textile sensors to develop the final device [28].

**Figure 4:**
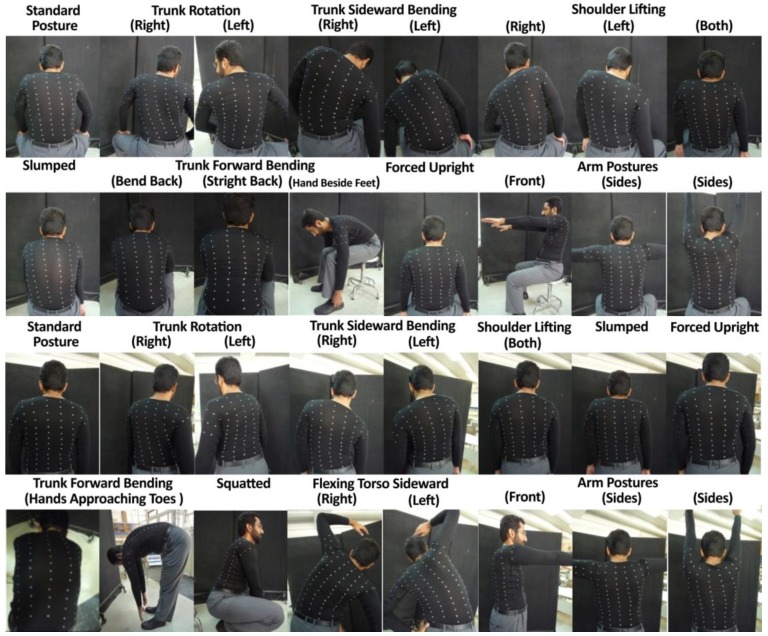
Thirty-two different trunk and shoulder movements used to determine the sensor placement configuration. The pictures were compared to the natural posture. In fact, the path of markers for each movement indicated the elongation direction of each sensor, which was used to determine the best sensor location.

To measure the 3D trunk angles by TMS, the BWSs needed to be placed so that they cover all possible trunk motions. We used a combination of the three methods mentioned-above. Ninety spherical markers were attached to a stretchable shirt [28]. Wearing this shirt, a healthy participant performed 32 different trunk and shoulder movements (Figure 4) similar to the study of Mattmann et al. [27], which covered all possible upper body movements. Captured pictures by a photo camera were analyzed qualitatively and compared to the natural posture. In fact, the path of markers for each movement indicated the elongation direction of each sensor, which was used to determine the sensor location.

**Figure 5:**
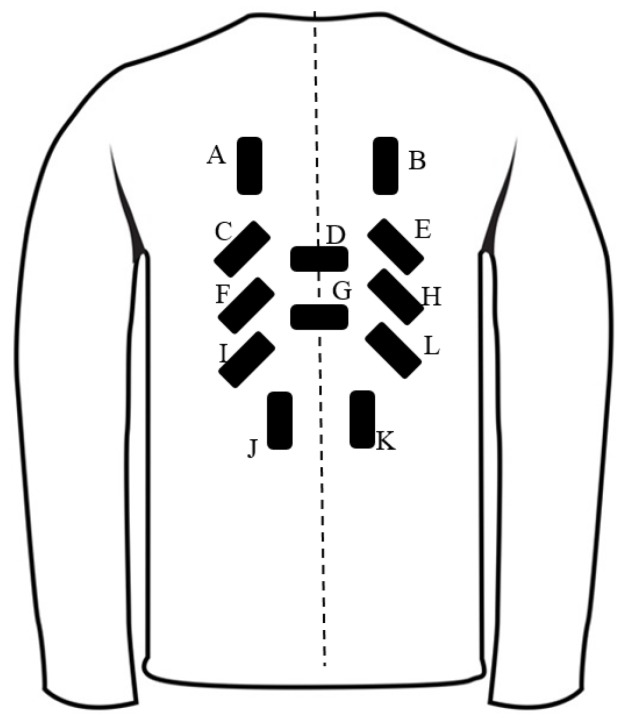
The identified positions of the BWSs as determined by the stretchable shirt. A, B, J, and K were included specifically for flexion/extension. Lateral bending was detected by C, F, I, E, H, and L. D and G were specified for axial rotations.

By analyzing the path of markers in the pictures above and considering the results of Mattmann and coworkers’ study, the potential orientations of the sensors’ placements were identified for trunk motions (Figure 5).

**Figure 6:**
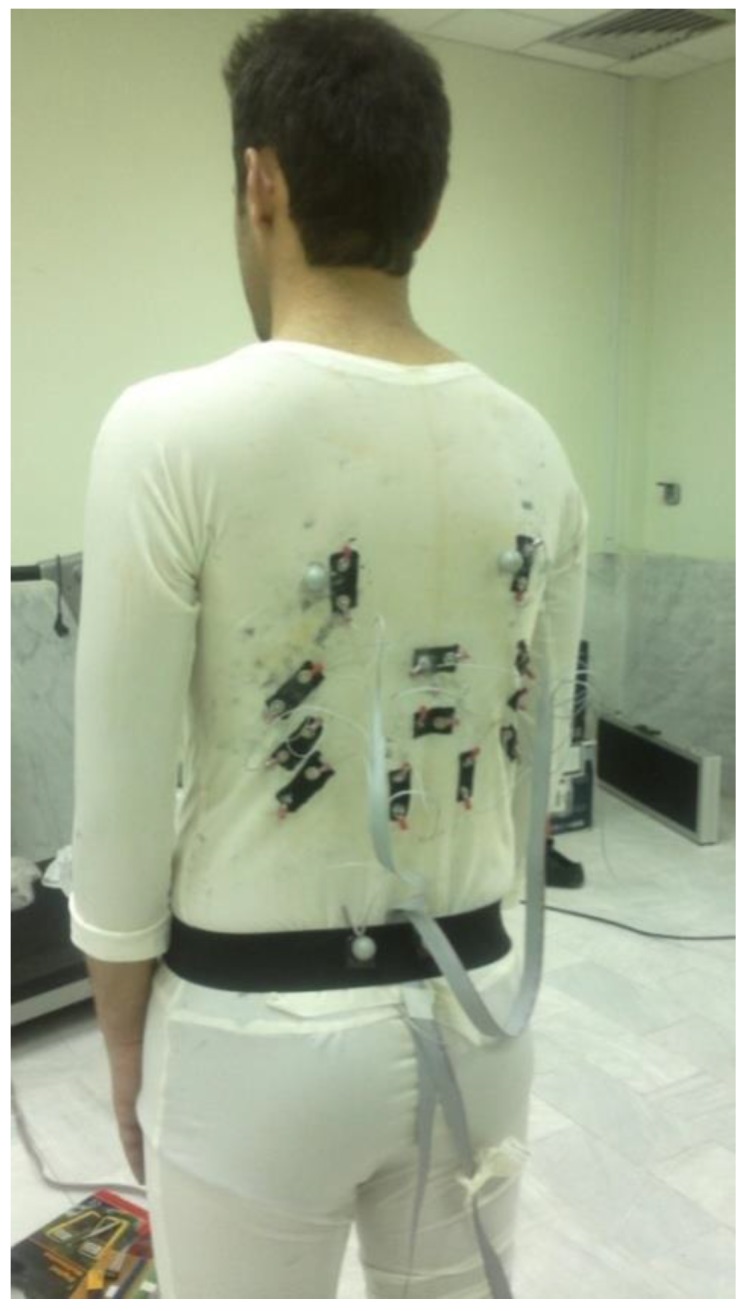
The final determined positions of the BWSs on clothing as manufactured. The clothing was fixed by a belt on the hip.

A, B, J, and K were included specifically for flexion/extension. Lateral bending was detected by C, F, I, E, H, and L. D and G were specified for axial rotations. Then the wearable trunk motion system (TMS) was manufactured using twelve body worn sensors (Figure 6).

#### 2.2.2. Calibration

Calibration process of the TMS contained two steps: BWS calibration and TMS calibration. First, several sensors had been placed in a zwickiLine materials testing machine (2.5/Z2.5, Zwick Co., Ulm, Germany) and the calibration characteristics were extracted. Regarding to the sensitivity, the gage factor (i.e., the sensitivity to strain) of the sensors was ~6 [29]. More details on this process are provided in our recent study [29]. A brief summary of these results is presented in Table 1.

In the second step, the TMS was calibrated using data sets obtained from a motion tracking system (Qualisys Inc.). For this purpose, an experiment was designed to calibrate the TMS for 3D anatomical movements.

#### 2.2.3. Experiment

**Figure 7:**
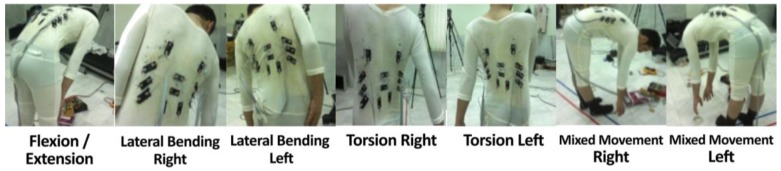
Seven tasks performed for the calibration of the TMS. Each participant performed five planar movements and two multiplanar movements.

Three angular trunk movements were measured using a seven-camera motion capture system (Qualisys Inc.), also simultaneously estimated using signals from 12 BWSs. The sampling frequency of motion capture system and BWSs were 100 Hz. Before performing the tests, retro-reflective spherical markers (1 cm in diameter) were placed on the anterior superior iliac spine (ASISs), posterior superior iliac spine (PSISs), the inferior angles of most caudal points of the two scapulas (SCAPs), sternum jugular notch (SJN), xiphoid process (XP), and the spinous process of the seventh cervical vertebra (C7). We modeled the pelvis and thorax segments by embedding a local coordinate system on each. The local coordinate system of pelvis was reconstructed using ASISs and PSISs based on coda pelvis model. Midpoint of C7 and SJN was considered as the thorax segment proximal joint; the midpoint between XP and midpoint between SCAPs was considered as the thorax segment distal joint. This coordinate is consistent with ISB recommendations [46]. Three healthy male participants were recruited to participate in this experiment with respective weight and height of 85, 78, and 65 kg; and 185, 175, and 165 cm. Participants performed seven different tasks; including five planar movements (the flexion-extension of the trunk, lateral bending to the left and right, both right and left axial rotations) and two multiplanar movements (see Figure 7 for more details). They repeated each task three times, in which ten cycles were performed in each repetition (i.e., 7 × 3 × 10 = 210 cycles per task).

#### 2.2.4. Motion Analysis Strategy

A common step signal was used to synchronize motion data and BWS signals. 3D coordinates of markers were filtered using a 4th order bidirectional Butterworth filter with a low-pass cutoff of 6 Hz. Flexion-extension, lateral bending, and axial rotations of the trunk were extracted from 3D filtered marker coordinates in different test conditions. Three-dimensional kinematics (flexion-extension, lateral bending, and axial rotation) of thorax and pelvis was extracted using the Euler–Cardan approach [47] programmed using MATLAB software (The MathWorks, Inc., Natick, MA, USA). The X-Y-Z cardan sequence was used to find the transformation matrix between thorax and pelvis local coordinate systems. Extracted 3D trunk angles were used to calibrate the TMS.

#### 2.2.5. Data Fusion Process

**Figure 8:**
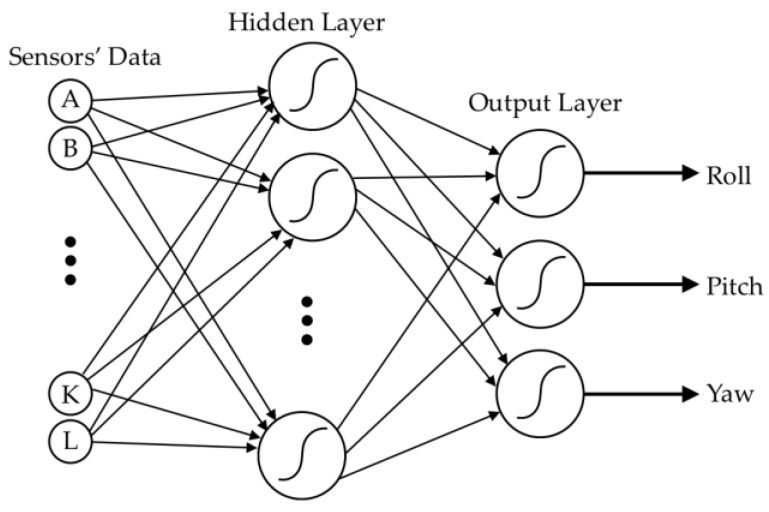
The block diagram of a neural network used to fuse inputs and map them to the outputs. There are 12 sensors’ signals and 3D angular position of the trunk as the inputs and outputs, respectively.

Each sensor produced the signals for all movements because its size was sufficiently large to detect each movement. In fact, all twelve sensors contributed to each movement. To determine the trunk angles from TMS, the raw BWSs’ signals were fused and mapped to the outputs of the Qualisys motion capture system using a two-layer feed-forward artificial neural network. The raw BWS signals were considered as 12 inputs of the neural network, while the outputs are the 3D angular position of the trunk (Figure 8).

Data fusion procedure was performed for each of the tasks (Figure 7) as well as the total movement, which is the combination of all of the tasks. First, for each of five simple planar tasks, a separate neural network was trained, evaluated, and tested, using the data that is obtained from all three participants. In these planar tasks, the movement is mainly caused by change in one of three angular positions. Thus, the accuracy of each neural network reflects the precision of the estimation of angular position in one dimension.

Second, since the complexity of two multiplanar movements would increase the generalization error, we used separate neural networks for different participants. In this step, six neural networks were used to estimate 3D angular position during multiplanar movements of three participants (three networks for right mixed movement and three networks for left mixed movement).

Finally, three neural networks, each for one participant were used to estimate angular positions during the combination of all of seven tasks. In each of these, 14 neural networks were incorporated in order to achieve the best network performance, and the most accurate one was selected afterward. Accuracy was defined as the root mean square error between the predicted and real angular positions.

A single procedure was used to train, evaluate, and test all of the 14 mentioned networks. In this approach, the data samples for each network was divided into three parts: training, evaluation, and testing phases. For training each network, 70% of the netwrok’s corresponding data samples were randomly selected and implemented in the network as the training phase. Levenberg-Marquardt based error backpropagation (EBP) algorithm [48] was used for updating weights and sigmoid activation function (for both hidden and output layers). The EBP algorithm that we used is an appropriate choice for training small and medium artificial neural networks [48]. Half of the remaining 30% data were randomly selected and utilized to determine network generalization and to break training when generalization stopped improving (evaluation). Finally, the remaining data (i.e., 15%) were used to test the trained network.

## 3. Results

Outcomes were presented in three sections, corresponding to the three parts of the network training process; planar movements, multiplanar movements, and the total accuracy of TMS for each participant. The accuracy of the TMS for planar and multiplanar movements was also reported briefly.

### 3.1. Planar Movements

Table 2 indicates the outcome of network training (i.e., the number of inputs/outputs, the number of neurons for data fusion; the number of samples for training, validation, and test performance; root mean square error (RMSE); and correlation coefficient) for planar movements including flexion/extension, left/right lateral bending, and left/right axial rotations. We tried different networks with various numbers of neurons in our classification algorithm, in order to achieve the best accuracy of network performance, then selected the most accurate one. Meanwhile, accuracy was defined as the root mean square error between the predicted and real outputs.

**Figure 9:**
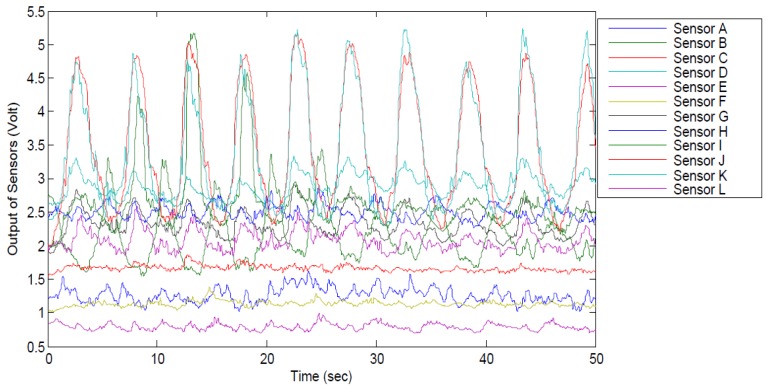
A sample diagram for ten cycles of flexion-extension performed by Participant 2. All sensors’ signals were adjusted to start from 1 V by potentiometers and some sensors were activated.

An illustrative sample output of the 12 BWSs including ten cycles of flexion/extension movement is illustrated in Figure 9. Using potentiometer, all sensors’ signals were adjusted to start from one volt. For this periodic movement, only some sensors were stretched and produced periodic signal.

### 3.2. Multiplanar Movements

The results of multiplanar movements for each participant are reported in Table 3. This Table contains the number of neurons for data fusion; the number of samples for training, validation and test performance; root mean square error (RMSE); and correlation coefficient.

**Figure 10:**
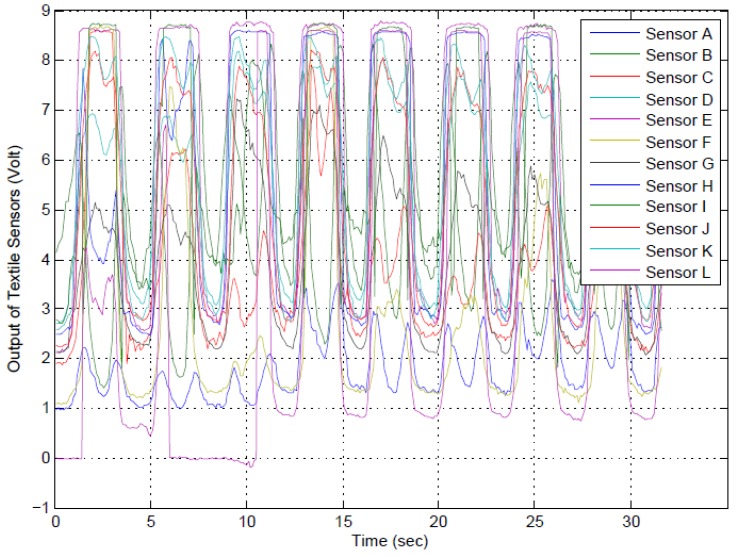
A sample diagram of 10 cycles for left multiplanar movement as performed by Participant 2. All sensors’ signals were adjusted to start from 1 V by potentiometers. The participant halted for a few seconds in extreme points. Therefore, a plateau can be seen for each cycle around 8.5 V.

Similar to the previous section, the output of the BWSs for ten cycles of left multiplanar movement as performed by Participant 2 is illustrated in Figure 10. All BWSs were fairly activated, and their signals changed during these multiplanar movements. All sensors’ signals were adjusted to start from one volt by potentiometers. The participant halted for a few seconds in extreme points. Therefore, a plateau can be seen for each cycle around 8.5 V.

### 3.3. The Total Accuracy for TMS

The accuracy of TMS for all movements of each participant including planar and multiplanar movements is presented in Table 4. The Table contains the number of neurons for data fusion; the number of samples for training, validation and test performance; root mean square error (RMSE); and correlation coefficient.

### 3.4. Summary of Results

**Figure 11:**
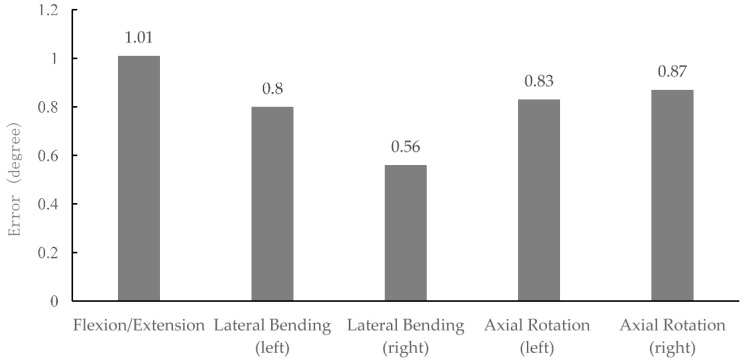
The accuracy of the system for planar movements for all participants.

The overview of TMS accuracy in planar movements for all participants is illustrated in Figure 11. The accuracy of the TMS for planar movements was found to be less than 1°.

**Figure 12:**
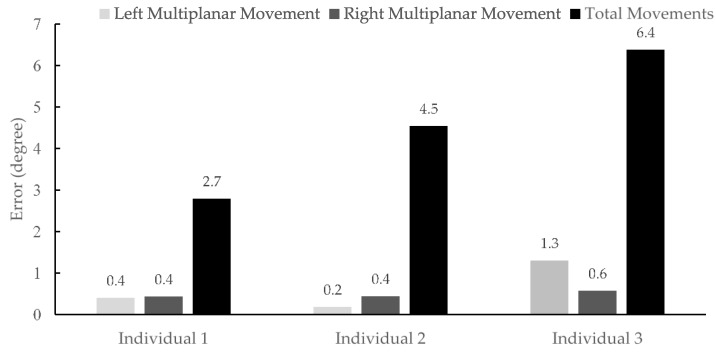
The accuracy of the TMS for multiplanar and total (planar and multiplanar) movements.

A summary of the accuracy for the TMS in multiplanar and total (both planar and multiplanar) movements for each participant is presented in Figure 12. The accuracies of the TMS reported as less than 1.3° for multiplanar movements. It can be observed that the accuracies for total (both planar and multiplanar) movements of Participants 1, 2 and 3 are 2.7°, 4.5°, and 6.3°, correspondingly.

## 4. Discussion

In this study, a trunk motion system (TMS) using printed body worn sensors (BWS) was designed and developed. TMS consisted of stretchable clothing, 12 printed Body-Worn Sensors (BWS), and electronic components. A shirt with 90 markers was used to determine optimal sensor locations and directions. Utilizing this shirt, 32 distinct movements were performed by a participant, and the path of markers for each movement was recorded by a photo-camera. Through qualitative analyses of this pictures, the position and elongation direction of each sensor were determined. Results of this experiment were compatible to the outcome reported by Mattmann et al. [28], where they used a motion tracking system to determine the locations of sensors by measuring the respective strain patterns [28]. Nevertheless, there were also other parameters that we needed to take into account, for example, the usability subjects, users’ privacy issues, and the type of garment [49,50,51]. Therefore, we may not conclude that the placement and number of sensors are optimal. Consequently, the suggested labels and positions of the sensors in Figure 5 were only potential location for the sensor placement. Finally, TMS was calibrated, and neural network technique was used to extract the motion data from the sensor signals.

One of our goals was to assess the accuracy of TMS system, also compare it to other existing systems. In fact, TMS accuracy in planar movements was determined to be less than 1° (Figure 4). This accuracy may help the utilization of TMS for specific applications related to the orientation of trunk in a planar dimension. TMS can also be a potential alternative for Lumbar Motion Monitor (LMM) [52], in 3D measurements of trunk movements, particularly since LMM is a bit bulky, also heavier than the TMS. The weight and size are two important factors which cause many limitations in practical situations/applications [11,12].

Few wearable devices have been developed to monitor the human motion. Tormene et al. [35] developed a wearable device and showed that it measured the trunk movement in the sagittal plane as accurate as IMUs. Other studies have used different kinds of textile sensors for different parts of the body. However, they mostly measure a single degree of freedom, and were not utilized for complex joints (e.g., trunk). In addition, their accuracy of kinematic estimation was not as good as the TMS in planar movement. For example, Tognetti et al. [40] developed a wearable goniometer to measure the knee flexion. They used a double-layer knitted piezoresistive technique to build their sensor with the accuracy of 5.3°. Szelitzky et al. [53] reviewed several low-cost displacement sensors and compared their performance in biomedical applications. Error of their measurements for different parts of the body was larger than TMS (i.e., 3.5° for hip and knee joints, 2° for knee angle (planar movement), and 11° for fingers angle). Furthermore, Tognetti et al. showed their porotype wearable sensor manufactured by knitted piezoresistive fabric measured the knee angle within 5° accuracy [40]. While accuracy of measurement was better in our system, it was not similar for all three participants. In fact, TMS outcome was more accurate for Participant 1 (~2.7° for both planar and multiplanar movements). This might be related to the fact that the clothing was tailored only for Participant 1. Using sized clothing for each participant is recommended for future studies.

It is notable that other systems such as inertial measurement units [17,18] may accurately measure the orientation during dynamic motions. However, they may not be utilized in widespread applications, since environmental conditions such magnetic fields can adversely affect their performance. Further, the attachment of external sensing elements on the body (e.g., IMU) may have some limitations for some application. First, the rigid object on the garment may decrease the comfortability and restrict the activities of daily life. Second, electronics components on the garment may not be washable. Third, the size and weight of the external objects may alter the usability of the product. Finally, attaching external devices changes the appearance of the garment, and this may offend wearers especially for patients with assistive or monitoring devices. The comparison between the TMS and inertial measurement unit (IMU) demonstrated a better usability for TMS system in some applications (Table 5).

One of the limitations of this study is the movement between the clothing and the skin of the human body. The calibration process with more repetitions of the tests may help to address this concern, especially since neural network algorithm (that used to train the network) utilized the data from calibration process that also included similar errors. We also modeled the trunk as one segment. One segment thorax limited our analysis, therefore, we need to model a multi-segment thorax in the future study. The best placement for the markers is also on the skin. However, if we have to cut the fabric, the response of textile sensors could have been changed, which could adversely affect the reliability of the results. Another limitation is related to several wires which were used to transfer the sensors signals to the electronic board. In the future, the wires will be replaced with conductive thread that will be part of the cloth. Furthermore, the optimal number and placement of sensors are still unknown, especially for the trunk as a complex segment. To address this, an optimization strategy may be helpful as a more accurate method [55]. Moreover, customized systems should be used for each participant to improve the systems accuracy for each individual. Another future direction of research is to test the system in more practical situations such as clinical applications and occupational exposure assessment. Further, system responsiveness to distinguish between different conditions such as healthy and disable movements, or novice and expert task accomplishment in different environments can be examined.

Among the advantages of TMS is the error compensation in Yaw direction of an IMU. This system can be aggregated to the data fusion algorithm as another source to enhance the system accuracy, particularly in the proximity to ferromagnetic objects. As another application, TMS can be used in the behavioral monitoring of daily activities. It may also have acceptable accuracy for human movement classification for daily activities, especially since we have twelve sensitive sensors, each can be considered as a source of information related to various activities. Finally, TMS can be useful for a broad application in many disciplines such as clinical movement analysis, ergonomic studies, sports biomechanics, and rehabilitation applications.

Smart textile sensors and systems were utilized in several areas such as sport, medical, gaming, military, and aerospace [56]. Our long-term goal is to investigate the accuracy of the TMS in activities of daily life, healthcare, and occupational scenarios. For these purposes, lab-based experiments will be completed to determine how accurately the TMS can detect several basic postures (sitting, standing, etc.) and diverse physical activity types (e.g., walking and running) in the mentioned settings. Outcomes of current study may facilitate integrating all components of textile sensor systems into a garment for monitoring the physiological and physical health factors in near future.

## 5. Conclusions

In this study, a new approach was developed to determine trunk orientation using a wearable measurement system. The results showed that the movements were measured with accuracy ranging from 0.5° to 1° for planar movements and 2.7° for multiplanar movements. High accuracy of the TMS along with its lightweight enables it to properly monitor and measure the trunk orientations. TMS can have several applications, for example in clinical, biomechanical, and ergonomic studies to prevent injuries and monitor the intervention efficiency.

## Figures and Tables

**Table 1:** Calibration results of BWS [29].

Characteristics	Max Strain	Max Strain Velocity	Linearity Error	Hysteresis Error	Repeatability Error	Relaxation Behavior
Amount	50%	400 (mm/min)	2%	8%	7%	11%

**Table 2:** The outcome of network training for planar movements.

Test	Input	Output	Neuron		Samples	RMSE ^1^	R ^2^
Flexion/Extension	12	1	40	Training	1750	0.65	0.998
				Validation	375	1.14	0.994
				Test	375	1.01	0.995
Lateral bending (left)	12	1	40	Training	1750	0.46	0.999
				Validation	375	1.06	0.997
				Test	375	0.80	0.998
Lateral bending (right)	12	1	40	Training	1750	0.35	0.999
				Validation	375	0.65	0.998
				Test	375	0.56	0.999
Axial rotation (left)	12	1	40	Training	1750	0.85	0.999
				Validation	375	0.61	0.999
				Test	375	0.82	0.999
Axial rotation (right)	12	1	40	Training	1750	0.40	0.999
				Validation	375	0.79	0.998
				Test	375	0.87	0.998

^1^ Root mean square error; ^2^ Correlation coefficient.

**Table 3:** The results of network training for the multiplanar movements performed by each participant. The outcome of network training for planar movements.

Test	Input	Output	Neuron		Samples	RMSE ^1^	R ^2^
Left multiplanar movement for Participant 1	12	3	90	Training	874	0.05	0.999
				Validation	188	0.37	0.999
				Test	188	0.40	0.999
Right multiplanar movement for Participant 1	12	3	40	Training	700	0.24	0.999
				Validation	150	0.38	0.999
				Test	150	0.43	0.999
Left multiplanar movement for Participant 2	12	3	60	Training	688	0.06	0.999
				Validation	148	0.14	0.999
				Test	148	0.18	0.999
Right multiplanar movement for Participant 2	12	3	60	Training	1050	0.22	0.999
				Validation	225	0.39	0.998
				Test	225	0.44	0.998
Left multiplanar Movement for Participant 3	12	3	90	Training	1750	0.72	0.999
				Validation	375	1.23	0.997
				Test	375	1.30	0.996
Right multiplanar movement for Participant 3	12	3	90	Training	1750	0.35	0.999
				Validation	375	0.57	0.999
				Test	375	0.57	0.999

^1^ Root mean square error; ^2^ Correlation coefficient.

**Table 4:** The results of the accuracy for TMS by all movements including planar and multiplanar movements as performed by each participant. The outcome of network training for planar movements.

Test	Input	Output	Neuron		Sample	RMSE ^1^	R ^2^
Participant 1	12	3	60	Training	10,324	2.67	0.979
				Validation	2213	2.97	0.976
				Test	2213	2.8	0.977
Participant 2	12	3	60	Training	8738	4.14	0.973
				Validation	1873	4.51	0.970
				Test	1873	4.54	0.968
Participant 3	12	3	45	Training	11,024	5.94	0.938
				Validation	2363	6.22	0.933
				Test	2363	6.38	0.932

^1^ Root mean square error; ^2^ Correlation coefficient.

**Table 5:** Comparison between IMU and TMS.

	Sensitivity to Ferromagnetic Objects	Weight of System	Sensor’s Dimension
IMU [18,54]	Yes	1.9 kg	38 × 53 × 21 mm
TMS	No	≤200 g	20 × 40 mm
